# Early severe institutional deprivation is associated with a persistent variant of adult attention‐deficit/hyperactivity disorder: clinical presentation, developmental continuities and life circumstances in the English and Romanian Adoptees study

**DOI:** 10.1111/jcpp.12576

**Published:** 2016-06-06

**Authors:** Mark Kennedy, Jana Kreppner, Nicky Knights, Robert Kumsta, Barbara Maughan, Dennis Golm, Michael Rutter, Wolff Schlotz, Edmund J.S. Sonuga‐Barke

**Affiliations:** ^1^ Department of PsychologyDevelopmental Brain‐Behaviour LaboratoryUniversity of SouthamptonSouthamptonUK; ^2^ The Amy Winehouse FoundationLondonUK; ^3^ Department of Genetic PsychologyFaculty of PsychologyRuhr‐University BochumBochumGermany; ^4^ MRC Social, Genetic & Developmental Psychiatry CentreInstitute of Psychiatry, Psychology and NeuroscienceKing's College LondonLondonUK; ^5^ Max‐Planck‐Institute for Empirical AestheticsFrankfurt am MainGermany

**Keywords:** Adult attention‐deficit/hyperactivity disorder, institutional deprivation, Romanian adoptees, adult onset, longitudinal, adversity

## Abstract

**Background:**

Early‐life institutional deprivation is associated with attention‐deficit/hyperactivity disorder (ADHD) later in childhood and adolescence. In this article, we examine, for the first time, the persistence of deprivation‐related ADHD into young adulthood in a sample of individuals adopted as young children by UK families after periods in extremely depriving Romanian orphanages.

**Methods:**

We estimated rates of ADHD at age 15 years and in young adulthood (ages 22–25 years) in individuals at low (LoDep; nondeprived UK adoptees and Romanian adoptees with less than 6‐month institutional exposure) and high deprivation‐related risk (HiDep; Romanian adoptees with more than 6‐month exposure). Estimates were based on parent report using DSM‐5 childhood symptom and impairment criteria. At age 15, data were available for 108 LoDep and 86 HiDep cases, while in young adulthood, the numbers were 83 and 60, respectively. Data on education and employment status, IQ, co‐occurring symptoms of young adult disinhibited social engagement (DSE), autism spectrum disorder (ASD), cognitive impairment, conduct disorder (CD), callous‐unemotional (CU) traits, anxiety, depression and quality of life (QoL) were also collected.

**Results:**

ADHD rates in the LoDep group were similar to the general population in adolescence (5.6%) and adulthood (3.8%). HiDep individuals were, respectively, nearly four (19%) and over seven (29.3%) times more likely to meet criteria, than LoDep. Nine ‘onset’ young adult cases emerged, but these had a prior childhood history of elevated ADHD behaviours at ages 6, 11 and 15 years. Young adult ADHD was equally common in males and females, was predominantly inattentive in presentation and co‐occurred with high levels of ASD, DSE and CU features. ADHD was associated with high unemployment and low educational attainment.

**Conclusion:**

We provide the first evidence of a strong persistence into adulthood of a distinctively complex and impairing deprivation‐related variant of ADHD. Our results confirm the powerful association of early experience with later development in a way that suggests a role for deep‐seated alterations to brain structure and function.

## Introduction

Attention‐deficit/hyperactivity disorder (ADHD) characterised by developmentally inappropriate and impairing symptoms of inattention, impulsivity and hyperactivity is a childhood onset disorder with deleterious effects across the life span (Chorozoglou et al., 2015; Faraone & Biederman, 2005; Sonuga‐Barke & Taylor, 2015). It is estimated to affect around 5% of children and adolescents worldwide (Polanczyk, Salum, Sugaya, Caye, & Rohde, 2015). It is heterogeneous – with three clinical presentations defined in DSM‐5 (American Psychiatric Association, 2013): with predominantly inattentive (ADHD‐PI), predominantly hyperactive/impulsive (ADHD‐PHI) and combined types (ADHD‐CT) affecting around 30%, 10% and 60% of patients, respectively (Willcutt, 2012). ADHD frequently co‐occurs with conditions such as conduct, mood and anxiety disorders (Yoshimasu et al., 2012), as well as with learning (Plourde et al., 2015) and pervasive developmental difficulties (Jang et al., 2013). ADHD is associated with lower IQ scores (Frazier, Demaree, & Youngstrom, 2004) and is common in children with intellectual disability (i.e. Ahuja, Martin, Langley, & Thapar, 2013). Elevated rates of insecure attachment have been noted, although the direction of causation remains to be established (Thorell, Rydell, & Bohlin, 2012). ADHD is around 2.5 times more common in males than females in the general childhood population (Arnett, Pennington, Willcutt, DeFries, & Olson, 2015; Willcutt, 2012) – although ADHD‐PI is more common in girls (Biederman et al., 2005; Willcutt, 2012).

Although initially regarded as a child and adolescent condition, ADHD is now also established as an important source of mental ill health and impairment in adulthood (Garcia et al., 2012). While as few as 20% of patients with ADHD in childhood continue to meet the full child diagnostic criteria in adulthood, substantial continuity is observed in those who continue to experience ADHD‐related impairment and subthreshold symptoms (Faraone, Biederman, & Mick, 2006). Comorbidities remain common but take on an adult form with antisocial personality disorder, mood and substance use disorders especially common (Rasmussen & Levander, 2009). Males and females appear to be more equally represented in the adult ADHD population (Matte et al., 2015).

In keeping with its high heritability (Larsson, Chang, D'Onofrio, & Lichtenstein, 2014), current aetiological models emphasise the role of genetic factors in ADHD (Thapar, Cooper, Eyre, & Langley, 2013). Historically, the role of prenatal environmental exposures such as those related to maternal smoking during pregnancy (Obel et al., 2016) and adverse intra‐uterine environments, marked by low birth weight, have also been investigated (Pettersson et al., 2015). Recent longitudinal studies confirming the link between ADHD and socioeconomic status (SES) suggest a role for postnatal social factors (Larsson, Sariaslan, Långström, D'Onofrio, & Lichtenstein, 2014; Russell, Ford, Rosenberg, & Kelly, 2014). However, such effects are nonspecific, marking, as they likely do, a myriad of adverse environmental exposures and familial genetic risks (Nigg & Craver, 2014). The same interpretational challenge is presented by studies linking ADHD to family conflict and maltreatment (McMillen et al., 2005; but see Harold et al., 2013). To date, the most compelling evidence for a predominantly social/environmental pathway to ADHD comes from studies of children exposed to nonfamily‐related adversity (as found, e.g., in institutional settings) and then placed with adoptive or foster families (van IJzendoorn et al., 2011). Elevated levels of inattention and hyperactivity/impulsivity have been reported in different institutionalised populations (Loman et al., 2013; McLaughlin et al., 2010, 2014; Roy, Rutter, & Pickles, 2004; Wiik et al., 2011), with effects growing stronger as a function of duration of institutional care and severity of deprivation experienced (Merz & McCall, 2010).

Evidence from the English and Romanian Adoptees (ERA) study is especially compelling in this regard (Rutter, Sonuga‐Barke, & Castle, 2010). The ERA has followed a group of children who lived for up to the first three‐and‐a‐half years of their lives in extreme deprivation in institutions in Romania, and then were adopted by families living in the United Kingdom soon after the fall of the Ceaușescu regime in 1989. The conditions in the institutions varied from poor to appalling, with little or no personalised care or social or cognitive stimulation. Hygiene and food were also badly compromised. The initial effects of deprivation, seen immediately postadoption, were profound and generalised, with extreme growth stunting and developmental delay (Rutter, 1998). By the age of 6 years, there was evidence of substantial developmental and physical growth catch‐up for many children indexed against a comparison group of nondeprived UK adoptees (O'Connor, Rutter, Beckett, Keaveney, & Kreppner, 2000). However, a substantial minority of children displayed patterns of impairment in rather specific but overlapping domains (Kreppner et al., 2010; Kumsta et al., 2010), described previously as deprivation‐specific problems (Rutter et al., 2010): quasi‐autism (Rutter et al., 2007), disinhibited attachment (Rutter et al., 2007) and cognitive impairment (Beckett et al., 2006). There was in each case a relationship between time spent in the institutions and severity of impairment – with a marked step‐wise increase in problem severity for individuals who had spent more than 6–9 months in the institutions that emerged most clearly in assessments carried out at ages 11 and 15 years. In fact, those individuals in the institutions for less than 6 months were in many ways indistinguishable from typically developing peers (Kreppner et al., 2007).

Many adoptees also displayed clinically elevated levels of ADHD symptoms at all follow‐ups – again showing the characteristic step‐wise increase with duration of deprivation (Kreppner, O'Connor, Rutter, & the ERA Team, 2001; Stevens et al., 2008). At age 15 years, individuals who experienced more than 6‐month deprivation were four times more likely to meet DSM‐IV diagnostic criteria for ADHD than those with less than 6‐month deprivation (16% vs. 4%) (Stevens et al., 2009). There were a number of features that appeared to distinguish deprivation‐related from nondeprivation‐related ADHD: the sex difference was less marked; there was an absence of comorbid conduct problems, but high levels of social disinhibition and autistic features; and neuropsychological impairment was unusually severe (Sonuga‐Barke & Rubia, 2008).

In this article, we provide the first evidence relating to the persistence of deprivation‐related ADHD into early adulthood using data from the recently completed ERA young adult follow‐up assessment carried out when the adoptees were aged 22–25 years. Our research questions were as follows:

1. Does a history of extended deprivation in institutions continue to place adoptees at risk for ADHD symptoms in early adulthood and, as previously found, does this manifest as a step‐wise increase in those experiencing more than 6‐month deprivation?
2. Is there a drop in the proportion of adult cases meeting full DSM criteria similar to that seen in typical ADHD clinic samples?
3. Is there a distinctive distribution of the three clinical presentations of ADHD, and does this change between adolescence and early adulthood?
4. If there are new onset cases in young adulthood, are they different from persistent cases?
5. Do equal number of males and females still meet ADHD criteria?
6. Is there anything distinctive about the pattern of co‐occurring disorders?
7. Is deprivation‐related ADHD associated with adult life underachievement and reduced quality of life (QoL)?

## Methods

### Participants

One hundred and sixty‐five Romanian adoptees and 52 comparison UK adoptees with no history of deprivation, and their families, initially entered the study in the mid‐1990s. For the purposes of the current analysis, we split the sample into two groups (Rutter et al., 2010). The first (LoDep) combined the UK comparison group and Romanian children who had less than 6‐month institutional deprivation. This group included a number of individuals who were adopted straight from their biological families. This LoDep group was contrasted with Romanian adoptees who experienced between 6‐ and 43‐months institutional deprivation (HiDep). At age 15, some outcome data were available for 199 participants [110 LoDep (including 48 UK adoptees) – 43.6% females; and 89 HiDep – 57.3% females]. By the young adult follow‐up (ages 22–25 years), this dropped to 164 [92 LoDep (including 42 UK adoptees) – 46.7% females; and 72 HiDep – 54.2% females]. The average age at young adult follow‐up for the UK comparison group was 23.2 (22–25, *SD* = .77) years and for the Romanian adoptees 23.6 (22–26, *SD* = .81) years. A comparison of age 15 characteristics of those that dropped out in young adulthood and those that remained in the study provided no evidence of selective attrition. The proportion of HiDep and LoDep cases was similar at the two ages, and there was no difference between those remaining in the study at the young adult follow‐up and those dropping out in terms of sex of child, age, IQ, or the proportion of cases with deprivation‐related problems at age 15 years (data available from authors).

### Measures

The ERA study included a wide range of measures at the adolescent and young adult follow‐ups. Only measures relevant to the current analysis are described here.

### Attention‐deficit/hyperactivity disorder

#### General strategy

ADHD and associated impairment were assessed using different instruments in mid‐adolescence and early adulthood. On the basis of these measures, we extracted common ADHD indices using the same reporting source (i.e. parent) and, as far as possible, equivalent thresholds for symptom presence and impairment (i.e. DSM‐5 childhood criteria). Our primary analysis used a categorical DSM‐5 definition of ADHD, allowing us to anchor our observations against clinical norms. We supplemented this with group comparisons of counts of symptoms of inattention and hyperactivity/impulsivity.

#### Adolescence

Data on ADHD were collected from parents as part of a modified Child and Adolescent Psychiatric Assessment (CAPA) interview (Angold & Costello, 2000). This is a well‐validated semi‐structured interview covering a range of psychiatric disorders assessed over the last 3 months. Information was collected about the presence of nine of the 18 DSM‐5 ADHD symptoms – 3 relating to inattention, 3 to hyperactivity and 3 to impulsivity (list of items symptoms available from authors). Each symptom was coded on a 0–3 severity scale by trained interviewers. Following the standard approach, a symptom was judged present when a score of 2 (present in at least two activities) or more was recorded. The criteria for presence of ADHD were met if at least 2 of 3 symptoms of inattention (ADHD‐PI), 4 of 6 symptoms of hyperactivity and/or impulsivity combined (ADHD‐PH/I) or both (ADHD‐CT) were reported along with ‘definite’ levels of impairment in daily functioning. These thresholds represented prorated equivalents of the full DSM‐5 18‐item childhood criteria.

#### Young adult

Parent ratings for all 18 DSM ADHD symptoms were collected using the Conners Comprehensive Behaviour Rating Scale (CBRS; Conners, Pitkanen, & Rzepa, 2011). This is a well‐validated scale covering a wide range of mental health and developmental disorders of childhood and adolescence. The version included items adapted for use with young adults following permission from the copyright holders. Items were rated in terms of ‘never, seldom’ to ‘very often, very frequently’ over the last month on a scale of 0–3. Presence of a symptom was coded using the standard approach (a score of 2 (often) or higher). To optimise the equivalence between assessment waves and to allow a like‐for‐like comparison over time, the current analysis of adult ADHD was based on the same nine symptoms that were available in the adolescent dataset. The same categorical thresholds were employed as in adolescence, and a rating of ‘always’ in at least two settings was required for impairment. The subscales for inattention and hyperactivity/impulsivity both had acceptable reliability, with Cronbach's alphas of .76 and .80, respectively.

#### Histories of ADHD symptoms in childhood

Estimates of ADHD symptoms were also available at ages 6 and 11 years, based on the three inattention/overactivity items of the well‐validated Rutter scale (Elander & Rutter, 1996). Parents rated each item on a scale of 0 (does not apply) to 2 (certainly applies).

### Co‐occurring developmental and mental health problems

#### Autism spectrum disorder

The Social Communication Questionnaire (SCQ; Rutter, Bailey, & Lord, 2003) was completed by parents at all assessment waves. It is a widely used and clinically validated 35‐item screen for autism spectrum disorder (ASD) symptoms that maps onto DSM diagnostic criteria. To ensure its developmental appropriateness in young adulthood, our analysis was based on a 15‐item version with five items from each scale (Social Reciprocal Interaction; Communication and Repetitive and Stereotyped behaviours; items available from authors). Items from the original were dropped on the basis of their distribution in the LoDep group because they were (a) too commonly endorsed in young adulthood and/or (b) showed a substantial increase between age 6 years and young adulthood – patterns inconsistent with items being considered markers of a serious/rare neurodevelopmental condition. Items were rated as either 0 for ‘No’ or 1 for ‘Yes’.

#### Disinhibited social engagement

This was assessed during both the mid‐adolescent and young adult assessment waves using three questions asked to parents based on those previously asked at age 11 and 15 (Kreppner et al., 2010; Kumsta et al., 2010; Rutter et al., 2007). The interview questions were as follows: ‘Seems too friendly with strangers or too eager to approach strangers?’ ‘Makes very personal comments or asked intrusive questions of others they've just met?’ and ‘Seems unaware of social boundaries, or the closeness of interaction with whom they are not familiar?’ Each question was rated on a 0–2 scale – with 0 representing ‘no evidence of disinhibition’ and 2 ‘definite evidence of disinhibition’.

#### IQ

A shortened version of the WASI (two‐subscale version, Wechsler, 1999) were administered in early adulthood.

#### Mood and conduct problems in young adulthood

Generalised anxiety, major depression and conduct disorder (CD) were assessed by the CBRS using standardised *T*‐scores. These ratings were based on self‐report (American Psychiatric Association, 2006). Levels of callous‐unemotional (CU) traits were examined using the parent report Inventory of Callous‐Unemotional traits (ICU; Essau, Sasagawa, & Frick, 2006), which measures the affective personality features of psychopathy. It contains 24 items assessed on a 0‐ to 3‐point Likert scale with higher scores reflecting increased levels of callous‐unemotional traits.

#### Quality of life

The Satisfaction with Life Scale (Diener, Emmons, Larsen, & Griffin, 1985) is a widely used, reliable and well‐validated self‐report measure of perceived QoL. On a scale from 1, strongly disagree, to 5, strongly agree, participants were asked whether they see their current life as ideal; are satisfied with life; would live life again in the same way; find life excellent; and think they have secured the important things in life. QoL is measured by a single sum score, with higher scores indicating better perceived QoL.

#### Young adult functioning

Key indicators of young adult functioning were derived from young adult and parent reports. These were currently ‘being unemployed’ and ‘having lower educational achievement’ (i.e. GCSEs or less). These were coded in a binary form (0 doesn't apply, 1 applies).

#### Adoptive family SES

This was based on data on parents’ occupation at the age 15 follow‐up (Rutter et al., 2010). Families were divided into high and low SES groups based on the Registrar General's classification (General Register Office, 1971). Manual and unskilled occupations were classified as low SES and skilled, managerial/technical and professional occupations as high SES.

#### Subnutrition at entry to United Kingdom

Many of the adoptees entered the United Kingdom severely subnourished. Consistent with our previous approach (Kumsta et al., 2010), we defined subnourishment as a weight more than 1.5 standard deviations below the UK norm.

#### Clinical engagement

Information about lifetime history of ADHD treatment was gathered from parents and young people during service use interviews at ages 6, 11, 15 and during the young adult follow‐up.

### Procedure

Ethical approval was obtained from the University of Southampton Research Ethics Committee. Informed consent was received from all participants. Assessments were carried out during face‐to‐face interviews in participants’ homes. Questionnaires were completed online or returned via the post.

## Results

### Preparatory analysis

Data on ADHD symptoms were available for 194 individuals in adolescence and 143 individuals in young adulthood. To check the validity of combining the UK and Romanian adoptees with less than 6‐month exposure to institutional deprivation, we compared ADHD rates in these two groups. Rates in both groups were similarly low in both adolescence [4.3% vs. 6.6% – χ^2^(1) = 0.27, *p* = .61] and early adulthood [2.8% vs. 4.7% – χ^2^(1) = 0.19; *p* = .66]. There was also no difference in the ADHD rates for the Romanian adoptees experiencing 6–24 months and over 24 months of institutional deprivation [adolescence: 21.4% vs. 16.7% – χ^2^(1) = 0.31; *p* = .58; young adulthood 34.4% vs. 23.1% – χ^2^(1) = 0.88, *p* = .35]. This pattern was replicated when continuous symptom counts rather than ADHD categories were used. The LoDep group contained a number of individuals (17 in adolescence and 11 in young adulthood) who were adopted straight from family homes. These individuals did not differ from the under 6‐month institutionalised group in terms of adolescent and young adult ADHD levels (*p*s > .31).

### ADHD persistence and continuity

**Figure 1:**
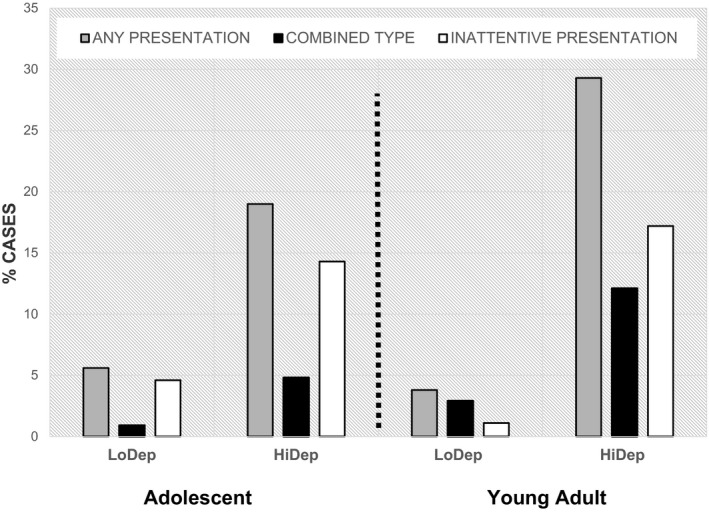
The proportion of ADHD cases by presentation type in the HiDep and LoDep groups. LoDep – UK comparison group plus Romanian adoptees with less than 6‐month exposure to deprivation; HiDep – Romanian adoptees over 6‐month deprivation

Figure 1 compares the proportion of individuals meeting ADHD thresholds in adolescence and early adulthood in the LoDep and HiDep groups. The effects of deprivation on ADHD appeared stronger in early adulthood than adolescence, with a risk ratio of 1:3.4 for the former and 1:7.7 for the latter. The same figures when only individuals with data at both time points were included were 1:2.5 and 1:7.8, respectively. A generalised estimating equations model tested effects of group (LoDep vs. HiDep) as a between‐subject variable and assessment age (adolescent vs. early adult) as a within subject variable; Sidak‐corrected pairwise comparisons between groups and assessment age were tested based on estimated marginal means. ADHD was significantly more frequent in HiDep group at both assessment waves (adolescent: difference = .14; *SE* = .048; *p* = .024; young adult: difference = .26; *SE* = .061; *p* = .001; Figure 1), but the interaction of group by age did not reach statistical significance (*p* = .12). There was also a high level of continuity at the individual case level with 70% of adolescent ADHD cases continuing to meet full diagnostic criteria in adulthood. ADHD‐PI predominated at both follow‐up points. In adolescence, 75% of ADHD cases had a predominantly inattentive presentation while this declined to 59% in the young adult group. When young adult identification was based on the full 18 DSM‐5 ADHD symptoms, the rates of adult ADHD of any type in the LoDep and HiDep groups were 4.7% and 26.7%, respectively, compared to 3.8% and 29.3% with the restricted nine‐item‐based estimate. For the predominantly inattentive presentation only, the rates were exactly the same for both versions, with 2.4% of the LoDep group and 18.3% of HiDep cases meeting criteria using both versions [χ^2^(1) = 11.00, *p* = .001]. The 9‐ and 18‐item scales identified the same cases on 93.3% of occasions [χ^2^(1) = 38.20, *p* < .001], for the predominantly inattentive presentation, and 93.8% [χ^2^(1) = 44.28, *p* < .001] for any presentation.

**Figure 2:**
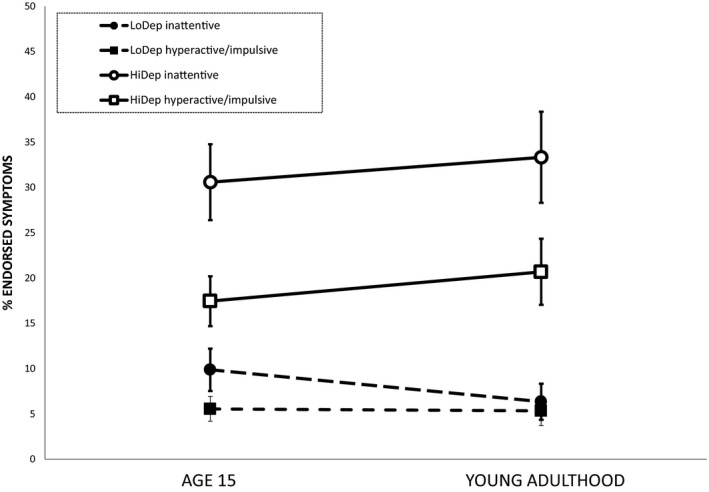
The proportion of symptoms of inattention and hyperactivity/impulsivity as a function of duration of deprivation and across time. Inattention was based on the three items, and hyperactivity/impulsivity, the six symptoms in common at on the parent‐rated CBRS. LoDep – combined UK adoptees with Romanian Adoptees with less than 6 months of deprivation: HiDep – combined all Romanian Adoptees with over 6 months of deprivation

Figure 2 plots the change between adolescence and young adulthood in terms of symptom counts for inattention and hyperactivity/impulsiveness separately. ANOVAs with group as a between‐subject variable and age as a within‐subject variable demonstrated a main effect of deprivation status. The HiDep group had significantly elevated levels of both inattention and hyperactivity/impulsivity at both ages, *F* (1, 136) = 30.32, *p* < .001, and *F* (1, 135) = 21.05, *p* < .001, respectively. There was a slight increase in the number of both symptom types across the age transition. but the interaction between group and age did not reach significance, *F* (1, 136) = .45, *p* = .51, and *F* (1, 135) = 1.04, *p* = .31. Consistent with the preponderance of ADHD‐PI, inattention symptoms were approximately 50% more likely to be endorsed than hyperactivity/impulsivity symptoms in the HiDep group.

### Persistent versus adult onset ADHD cases

There were nine ‘adult onset’ and nine adolescent‐adult ‘persistent’ cases. Table 1 compares these two groups with young people who did not meet ADHD cut‐offs at either age (no‐ADHD) on childhood and adolescent levels of ADHD and young adult characteristics. Onset cases had a history of elevated ADHD symptoms at 6, 11 and 15 years relative to the no‐ADHD cases. In general, the persisters had higher levels of symptoms of anxiety, depression, CU traits, disinhibited social engagement (DSE), and lower IQ compared to the onset group, although a lack of statistical power meant that these effects did not reach significance.

**Table 1:** A comparison of characteristics between persisters, adult onset and no‐ADHD cases for the whole sample

	Developmental profile			Group contrasts		
	No ADHD (N) (*n* = 108)	Onset (O) (*n* = 9)	Persisters (P) (*n* = 9)	N versus O	N versus P	O versus P
ADHD in childhood and adolescence						
At age 6	0.50 (0.50)	1.22 (0.37)	1.29 (0.47)	***t*** **(114) = 4.26, ** ***p *** **< .001**	***t*** **(114) = 4.61, ** ***p *** **< .001**	*t*(16) = .35, *p* =* *.73
At age 11	0.47 (0.53)	1.03 (0.47)	1.50 (0.50)	***t*** **(108) = 3.05, ** ***p = *** **.003**	***t*** **(108) = 5.58, ** ***p *** **< .001**	*t*(16) = 2.03, *p = *.06
At age 15	0.51 (1.22)	2.11 (1.62)	5.33 (1.80)	***t*** **(115) = −3.69, ** ***p *** **< .001**	***t*** **(115) = −10.96, ** ***p *** **< .001**	***t*** **(16) = −3.99, ** ***p = *** **.001**
Young adult outcomes						
ADHD (IA)	1.11 (1.76)	7.0 (2.24)	7.78 (1.30)	***t*** **(115) = −7.70, ** ***p *** **< .001**	***t*** **(115) = 11.07, ** ***p *** **< .001**	*t*(16) = −0.90, *p = *.38
ADHD (HYP/IMP)	0.52 (1.01)	3.33 (2.60)	4.33 (2.60)	***t*** **(8.20) = −3.23, ** ***p *** **≤ 012**	***t*** **(8.20) = 4.38, ** ***p = *** **.002**	*t*(16) = −0.82, *p = *.43
Anxiety	54.05 (13.33)	59.75 (11.84)	70.67 (7.28)	*t*(93) = −1.17, *p = *.25	***t*** **(91) = −3.01, ** ***p = *** **.003**	*t*(12) = −1.98, *p = *.07
Depression	54.06 (13.97)	63.00 (11.64)	69.00 (11.80)	*t*(93) = 1.75, *p* = .08	***t*** **(91) = 2.56, ** ***p*** ** = .01**	*t*(12) = ‐.95, *p* = .36
CD	46.08 (10.78)	53.25 (14.45)	54.17 (7.25)	*t*(93) = 1.75, *p* = .08	*t*(91) = 1.80, *p* = .08	*t*(12) = ‐.14, *p* = .89
CU	25.63 (6.70)	34.75 (6.56)	37.03 (6.24)	***t*** **(104) = 3.71, ** ***p *** **< .001**	***t*** **(104) = 4.65, ** ***p *** **< .001**	*t*(14) = 0.71, *p* = .49
DSE	0.37 (1.04)	0.78 (1.09)	1.78 (1.98)	*t*(109) = 1.11, *p* = .27	*t*(109) = 2.10, *p* = .07	*t*(16) = −1.32, *p* = .21
ASD	0.84 (1.55)	3.59 (2.61)	2.34 (1.49)	***t*** **(8.52) = −3.11, ** ***p*** ** = .01**	***t*** **(107) = −2.79, ** ***p*** ** = .006**	*t*(16) = 1.24, *p* = .23
IQ	101.52 (14.84)	92.0 (10.71)	88.0 (17.80)	*t*(93) = 1.66, *p* = .10	***t*** **(93) = 2.29, ** ***p*** ** = .02**	*t*(12) = 0.51, *p* = .62
QoL	17.70 (5.24)	11.88 (5.22)	15.33 (4.46)	***t*** **(87) = 3.00, ** ***p*** ** = .003**	*t*(85) = 1.08, *p* = .28	*t*(12) = −1.30, *p* = .22

Figures in bold refer to significant effects. Where degrees of freedom have decimal places, corrected values have been used to account for unequal variances. ADHD symptoms at age 6 and 11 based on the Rutter parent scales and at age 15 the CAPA. ADHD (IA) = CBRS inattentive ADHD symptoms; ADHD (HYP/IMP) = CBRS hyperactive/impulsive ADHD symptoms; Anxiety = self‐rated CBRS *T*‐scores; depression = self‐rated CBRS *T*‐score; CD = conduct disorder self‐rated CBRS *T*‐score; CU = parent‐rated Callous‐Unemotional Traits score; DSE = disinhibited social engagement (formerly disinhibited attachment); ASD = parent‐rated Social Communication Questionnaire; QoL = self‐rated Satisfaction with Life score.

### Characteristics associated with ADHD in young adulthood

Table 2 compares the LoDep group with those in the HiDep group who met ADHD diagnostic criteria (ADHD+) and those in the HiDep group who did not meet ADHD thresholds (ADHD−) in terms of sex, prior treatment for ADHD and co‐occurring problems and early adult circumstances. There was no difference between the three groups in the ratio of males to females, with roughly similar numbers of each sex meeting diagnostic criteria for ADHD. ADHD+ was not associated with subnutrition at entry to the United Kingdom or with adoptive family social class. The ADHD+ group were more likely than both the ADHD− and the LoDep groups to have received treatment for ADHD (41%). After adjustment for multiple testing, the ADHD+ group had significantly higher levels of both ASD and CU traits than the ADHD− group. The ADHD+ group exhibited more DSE than the LoDep group. Levels of mood and conduct problems were highest in the ADHD+ group, but they did not differ significantly from the ADHD− group. Levels of conduct problems were particularly low based on *T*‐score estimates. The very high rates of unemployment (almost 90%) and low educational achievement (over 70%) in the ADHD+ group were significantly greater than in both other groups. Furthermore, QoL was lowest in the ADHD+ group.

**Table 2:** Demographic characteristics and clinical outcomes for LoDep and HiDep young adults with and without ADHD

	LoDep (*n* = 79)	HiDep		Group contrasts		
		ADHD− (*n* = 41)	ADHD+ (*n* = 17)	LoDep versus ADHD−	LoDep versus ADHD+	ADHD− versus ADHD+
Sex (% f)	42.9	53.7	58.8	χ^2^(1) = 1.43, *p* = .23	χ^2^(1) = 1.53, *p* = .22	χ^2^(1) = 0.13, *p* = .72
Low SES (%)	13.0	15.8	13.3	χ^2^(1) = 0.19, *p* = .66	χ^2^(1) = 0.00, *p* = .97	χ^2^(1) = 0.05, *p* = .82
Subnourished at entry to United Kingdom (%)	50.0	82.9	70.6	**χ** ^**2**^ **(1) = 11.71, ** ***p*** ** = .001**	χ^2^(1) = 2.31, *p* = .13	χ^2^(1) = 1.12, *p* = .29
Treated for ADHD (%)	6.7	12.2	41.2	χ^2^(1) = 1.22, *p* = .27	**χ** ^**2**^ **(1) = 17.99, ** ***p *** **< .001**	**χ** ^**2**^ **(1) = 6.15, ** ***p*** ** = .01**
Clinical characteristics (mean, *SD*)						
DSE	0.15 (0.63)	1.03 (1.58)	1.65 (1.84)	***t*** **(122) = 3.33, ** ***p*** ** = .002**	***t*** **(100) = 3.32, ** ***p*** ** = .004**	*t*(54) = −1.29, *p* = .20
ASD	0.99 (1.83)	1.59 (2.32)	4.91 (4.23)	*t*(57.33) = 1.38, *p* = .17	***t*** **(17.28) = −3.75, ** ***p*** ** = .002**	***t*** **(52) = −3.04, ** ***p *** **< .001**
IQ	102.68 (16.09)	96.0 (13.11)	93.27 (10.62)	*t*(105) = 2.02, *p* = .05	*t*(86) = 1.88, *p* = .06	*t*(39) = 0.62, *p* = .54
CD	46.38 (10.78)	48.45 (13.45)	51.36 (11.16)	*t*(101) = 0.83, *p* = .41	*t*(81) = 1.42, *p* = .16	*t*(40) = 0.64, *p* = .52
CU	25.99 (7.0)	26.73 (7.91)	35.82 (6.30)	*t*(114) = −0.51, *p* = .61	***t*** **(91) = 5.05, ** ***p *** **< .001**	***t*** **(51) = 3.97, ** ***p *** **< .001**
Depression	54.29 (13.96)	58.19 (14.97)	65.00 (12.60)	*t*(101) = −1.27, *p* = .21	***t*** **(81) = 2.40, ** ***p*** ** = .02**	*t*(40) = 1.34, *p* = .19
Anxiety	54.15 (13.63)	58.03 (14.03)	62.73 (11.86)	*t*(102) = 1.32, *p* = .19	*t*(82) = 2.00, *p* = .05	*t*(40) = −0.99, *p* = .33
QoL	16.92 (5.38)	18.19 (5.05)	14.00 (5.33)	*t*(97) = −1.06, *p* = .29	*t*(81) = 1.68, *p* = .10	***t*** **(36) = −2.28, ** ***p*** ** = .03**
Young adult functioning						
Unemployed (%)	12.0	24.4	88.2	χ^2^ = 3.30, *p* = .07	**χ** ^**2**^ ** = 45.96, ** ***p *** **< .001**	**χ** ^**2**^ ** = 19.97, ** ***p *** **< .001**
Low education (%)	26.4	31.7	76.5	χ^2^ = 0.39, *p* = .53	**χ** ^**2**^ ** = 15.96, ** ***p *** **< .001**	**χ** ^**2**^ ** = 9.74, ** ***p*** ** = .002**

Figures in bold refer to significant effects. Where degrees of freedom have decimal places, corrected values have been used to account for unequal variances. LoDep = combined UK adoptees with Romanian Adoptees with less than 6 months of deprivation; HiDep = combined all Romanian Adoptees with over 6 months of deprivation; SES = socioeconomic status based on family occupational status at age 15; subnutrition at entry to UK = proportion will weight 1.5 *SD*s below UK norms; ADHD− = HiDep individuals not meeting ADHD criteria; ADHD+ = HiDep individuals meeting ADHD criteria; DSE = disinhibited social engagement (formerly disinhibited attachment); ASD = parent‐rated Social Communication Questionnaire mean and *SD*; CD = conduct disorder (self‐rated CBRS *T*‐score); CU = parent‐rated Callous‐Unemotional Traits; depression = self‐rated CBRS *T* score; Anxiety = self‐rated CBRS *T*‐scores mean and *SD*; QoL = self‐rated Satisfaction with Life score; Low education = GCSEs or less.

## Discussion

The form of ADHD observed in the current study in individuals exposed to severe deprivation in early childhood shares features with ADHD in typical clinical populations. It is also different in important ways. First, compared with ‘typical’ ADHD, it appears to be unusually persistent across the transition from adolescence to adulthood – a pattern previously seen in the ERA sample across childhood and early adolescence (Stevens et al., 2009). This persistence was observed for both symptoms and impairment (a pattern differing from typical clinical populations where symptoms often remit to subclinical levels; Faraone et al., 2006). Symptomatic persistence was manifest at the group level, both in terms of ADHD categories and ADHD symptom counts. There was also strong continuity at the level of the individual. These data on persistence of risk are consistent with the notion that early exposure to severe adversity can have a powerful detrimental effect on long‐term mental health and well‐being (Rutter & O'Connor, 2004) – in this case, stretching from the very earliest years of life to adulthood. Crucially, these effects were found despite the deprived individuals having spent the vast majority of their lives, postexposure, in loving, supportive and well‐resourced families.

If true, the possibility raised by the current results, that deprivation‐related ADHD may represent a persistent variant of the condition, challenges current thinking in a number of ways. First, somewhat paradoxically, they suggest that ADHD established following early adversity may be less open to later‐operating genetic and environmental protective influences that can determine patterns of disorder offset in late childhood and early adolescence in nondeprived samples. Second, it seems to contradict the received wisdom, from developmental psychopathology, that the further one moves in time away from an initial risk exposure, the smaller is its impact on well‐being.

What is the root of this persistence of deprivation‐related risk? One very plausible hypothesis is that deprivation‐related ADHD results from early established deep‐seated neurobiological alterations, the persistence and severity of which are determined by the scale of the exposure and its timing (see Nelson, Bos, Gunnar, & Sonuga‐Barke, 2011 for a discussion). Testing this neurobiological hypothesis is beyond the scope of this study. It is subject of ongoing research, as are studies of epigenetic signatures of early deprivation (Kumsta et al., 2016).

One striking feature of the persistence of deprivation‐related risk was the number of apparently ‘new’ onset cases – those who met ADHD criteria in young adulthood but not in adolescence. There are at least two possible explanations for this pattern. First, it may be that these young people had a long‐standing liability to ADHD, which manifested itself only after the transition to adulthood. The underlying risk could have been held in check by the buffering effect of high functioning adoptive family environments. Alternatively, it could be exacerbated or activated, by additional stresses associated with adult life. In both cases, the effects of early adversity might only become apparent when individuals leave home and move away from these protective environments (examples of a sleeper effect). A second possibility is that this form of ‘adult onset’, ADHD is a different sort of condition to the childhood variety occurring, rather, as a secondary feature of other co‐occurring conditions. This possibility has been raised in relation to adult onset cases of ADHD generally (Moffitt et al., 2015).

We examined this question in two ways. First, we compared onset and persistent ADHD in terms of the presence of ADHD symptoms earlier in childhood. Interestingly, both groups had significantly elevated rates of symptoms at ages 6, 11 and 15 years. Second, we compared the two groups in terms of co‐occurring problems in young adulthood. While the comparison lacked statistical power, there was no evidence of greater levels of comorbidity in the adult/later onset group – if anything, the persisters had a more complex and severe presentation. Taken together these findings suggest that the adult onset group shared some neurodevelopmental origins with the persistent group but were perhaps less severely affected and complex in presentation – a pattern of results which seems more consistent with the sleeper effect hypothesis.

In addition to greater persistence, deprivation‐related ADHD in the current study had other characteristics that marked it out from more common forms of the condition. Four characteristics are particularly striking. First, and contrasting with the sex ratios found in population‐based epidemiological studies, it was equally common in males and females. Given that common risks (e.g. genetic variants) for ADHD seem to operate, in general, to the detriment of boys, this suggests that girls are more vulnerable to the risks associated with deprivation, at least in terms of ADHD. However, it must be remembered that the mortality rates were relatively high in the Romanian institutions and there is a possibility that the most vulnerable males suffered a disproportionate level of mortality, with only the more resilient males surviving (Kligman, 1998).

Second, while it is important to acknowledge that symptoms of hyperactivity and impulsivity were also elevated, there was a preponderance of the predominantly inattentive type following deprivation in the HiDep group. This may simply be a consequence of the high number of girls in the ERA ADHD population. However, there is evidence that executive functions are specifically compromised in institutionally deprived individuals – with deficits placing children specifically at risk for attentional rather than hyperactive and impulsive behaviours (Beckett, Castle, & Sonuga‐Barke, 2010; Colvert et al., 2008; Hostinar, Stellern, Schaefer, Carlson, & Gunnar, 2012; Loman et al., 2013; Pollak et al., 2010).

Third, deprivation‐related ADHD has a characteristic pattern of comorbidities marked by persistently high DSE, ASD and CU traits and low CD. In the past, based on data from the age 11‐ and 15‐year follow‐ups, we have speculated about the existence of an institutional deprivation syndrome, the core of which is DSE and QA with deprivation‐specific ADHD and cognitive impairment (CI) being distinctive common associated features (Kreppner et al., 2010; Kumsta et al., 2010). CU traits have not previously been regarded as an element of this deprivation‐specific pattern, but the current results, together with those from the age 15 follow‐up, suggest that we should consider extending the phenotype description (Kumsta, Sonuga‐Barke, & Rutter, 2012). Although this study was not designed to test this (and lacked sufficient statistical power to do so formally), the raised levels of ASD and DSE in the high‐risk ADHD group suggest a clustering of these problems in a way that is consistent with such a hypothesis. The reason for the very low levels of conduct disorder remains to be determined. The finding stands in sharp contrast to studies of both ADHD populations (Erskine et al., 2014) and samples exposed to maltreatment and social deprivation (Cecil, Viding, Barker, Guiney, & McCrory, 2014; Murray et al., 2015) where high levels of CD are very common. One possibility is that CD is rare because of the specific presentation of ADHD found in the ERA sample (i.e. more inattentive and more female). Second, it may be rare because of the role of specific aetiological factors – ADHD forms resulting from environmental exposures, compared to genetic factors, might be less pleiotropic in their expression. Third, and related to the discussion above, growing up in a well‐functioning family may buffer family‐related risks that typically provoke the development of CD in ADHD individuals – such as high expressed emotion and harsh discipline (Scott et al., 2010) or peer‐related deviance (Marshal, Molina, & Pelham, 2003). Indeed, Taylor (1999) has shown that conduct problems are most likely to develop in children with ADHD when they are exposed to high levels of negative expressed emotion, indicative of suboptimal parenting.

Given its distinctive putative aetiology, persistence, presentation and pattern of comorbidities, one might question whether the term ADHD should be applied to the problems of attention, and to a lesser extent, impulsiveness and hyperactivity, displayed by some of the HiDep group. However, a change in diagnostic label would not be justified from the perspective of the current phenomenological approach to diagnosis employed by the DSM, which recognises the heterogeneity of the condition and makes no specific exclusions regarding social aetiological factors or patterns of co‐occurring problems. Indeed, the value of distinguishing an institutionally deprived subtype of ADHD would rest on its ability to better predict prognosis and treatment response. While we now have some indication that it may represent an unusually persistent form, we know little about the impact of standard ADHD therapies. On balance, we have thus chosen to describe this as a deprivation‐related variant of ADHD.

The findings have a number of clinical implications. First, it is clear that deprivation‐related ADHD is associated with substantial clinical need, with poor long‐term outcomes (e.g. unemployment and poor educational attainment) and with reduced QoL. The clinical imperative is to ensure that early deprived individuals with ADHD get the specialist services they need and that these continue into adult life. In this regard, at around 40%, the lifetime rate of clinical engagement in the ERA sample was relatively high. More generally, the current findings highlight the importance of measuring early‐life exposures to adverse environments in children with ADHD during clinical assessments in order to properly address the especially persistent and complex nature of the problems such individuals present.

The current study had significant strengths including its prospective nature and the stratification of deprivation‐related risk to increase statistical power. There were also a number of limitations that need to be considered when interpreting its findings. One related to the way that ADHD symptoms were assessed. First, different approaches were used in adolescence and young adulthood to collect ADHD information – parental interview in adolescence and questionnaire in young adulthood. Second, information on only nine ADHD items was collected during the adolescent interview. We addressed these constraints by using a categorical approach to measuring ADHD – to reduce the impact of the different scaling properties of the two instruments on scores; by restricting the main young adult analyses to the same nine symptoms assessed in adolescence and adjusting the diagnostic thresholds accordingly; and by employing the same DSM childhood criteria at both ages (i.e. not employing the new DSM‐5 adult criteria). This approach led to plausible estimates of ADHD rates in the LoDep group. While we were initially concerned that use of questionnaires in the young adult sample would inflate rates, in fact the rates in the LoDep group were lower in the young adult assessment than in the adolescent assessment. Crucially, when rates of young adult ADHD were based on the full 18 items, they were very similar to those derived using the nine‐item scale. The level of agreement between the two versions was also high.

A second limitation concerned the level of attrition at the young adult assessment, which was much higher than the very low rates previously seen in the study. This, although not surprising given the age range of the young adults, limited the statistical power of the analysis, especially in the comparison of ADHD and non‐ADHD subgroups. Crucially, however, there was no evidence that attrition was selective, with those retained to young adulthood and those lost to the study after the age 15 follow‐up differing little on key measures. In addition, all the analyses reported in this article were conducted again only for those individuals with data at both ages and the effects did not change. A third limitation relates to the limited number of young adult measures available for analysis of co‐occurring difficulties. Unfortunately, measures of personality disorders and substance abuse were not available for analysis at the time of writing this article. A final limitation was that reports of impairments used to identify ADHD cases could not be tied specifically to ADHD symptoms and could have been a product of co‐occurring problems.

In summary, this study provides the first evidence of a strongly persistent and distinctively complex and impairing institutional deprivation‐related variant of adult ADHD. This highlights the powerful impact of early experience on later development in a way that potentially implicates deep‐seated neurobiological alterations. Clinical services need to be especially mindful of the need to ensure an effective transition from adolescent to young adult services in ADHD individuals exposed to early adversity.

**Key points**

- Early severe institutional deprivation is associated with adverse long‐term outcomes in a substantial minority of cases.
- In the English and Romanian Adoptees study, ADHD− is a characteristic feature of a pattern of deprivation‐related childhood and adolescent problems also including quasi‐autism, disinhibited social engagement, and cognitive impairment.
- This study provides the first evidence of a persistent variant of adult ADHD found in individuals exposed to severe institutional deprivation early in childhood.
- Deprivation‐related adult ADHD was distinctive in terms of frequency of the inattentive presentation, high proportion of females and co‐occurrence with disinhibited social engagement, ASD symptoms and CU traits.
- The strong persistence and complex nature of ADHD in adults exposed to severe early deprivation highlights the need to optimise continuity and cooperation between child and adult clinical services.
